# PD-L1 regulates genomic stability via interaction with cohesin-SA1 in the nucleus

**DOI:** 10.1038/s41392-021-00463-0

**Published:** 2021-02-25

**Authors:** Wen Zhang, Jiali Jin, Yanjin Wang, Lan Fang, Liu Min, Xinbo Wang, Lin Ding, Linjun Weng, Tan Xiao, Tianhua Zhou, Ping Wang

**Affiliations:** 1 Tongji University Cancer Center, Shanghai Tenth People’s Hospital of Tongji University, School of Medicine, Tongji University, Shanghai, China; 2 Department of Cell Biology and Cancer Institute of the Second Affiliated Hospital, Zhejiang University School of Medicine, Hangzhou, China; 3 School of Life Sciences and Technology, Tongji University, Shanghai, China; 4 Cancer Center, Zhejiang University, Hangzhou, China; 5 Department of Molecular Genetics, University of Toronto, Toronto, ON Canada

**Keywords:** Cell biology, Cancer

**Dear Editor,**

Programmed death ligand-1 (PD-L1) is a type 1 transmembrane protein and highly expressed in various cancers that binds to PD-1 on T cells, inhibits T cell activity and proliferation, facilitates cancer cells to escape T cell-mediated immune surveillance. Immunotherapies by PD-L1/PD-1 blockade have shown effectiveness against different cancer types and revolutionizes cancer treatment in the clinic. However, the response rate to anti-PD-L1/PD-1 antibody remains at about 15–30% as a single agent.^1^ Thus, there is more to understand regarding the function of PD-L1 in cancer.

A recent work shows PD-L1 plays essential roles in tumor autophagy, DNA damage response, and tumor glucose utilization via an intracellular mechanism.^2^ Moreover, several studies indicate that PD-L1 could be detected in the nucleus and probably related to drug resistance and self-renewal potential of cancer stem cells. However, the function of nuclear PD-L1 in tumor biology remains largely unknown.

Cohesin, a highly conserved ring-shaped protein complex including Scc1/RAD21, Scc3/SA (SA1 or SA2), SMC1, and SMC3, is thought to control genomic integrity by sister chromatid cohesion. Dysfunction of cohesin can indeed affect tumorigenesis by increasing genome instability. Mutations in SA1 and SA2 have been identified in many human cancers. Knockdown of SA1 shows increased cohesion defects, aneuploidy and inhibits cell proliferation.^3^

During study the role of PD-L1 in cancer cells, we surprisingly found that depletion of PD-L1 significantly increases chromosomal defects of cancer cells by measuring the anaphase bridge and lagging chromosomes during mitosis (Fig. 1a–c). Furthermore, we found that knockout of PD-L1 leads to an increase in aneuploidy (Supplementary Fig S1a, b). Furthermore, depletion of PD-L1 significantly impairs telomere cohesions, but had little effect on centromere cohesions (Fig. 1d–f; Supplementary Fig. S1c, d). These data suggest that PD-L1 is involved in the regulation of telomere cohesion, and may contribute to the maintenance of the genomic stability.

**Fig. 1:**
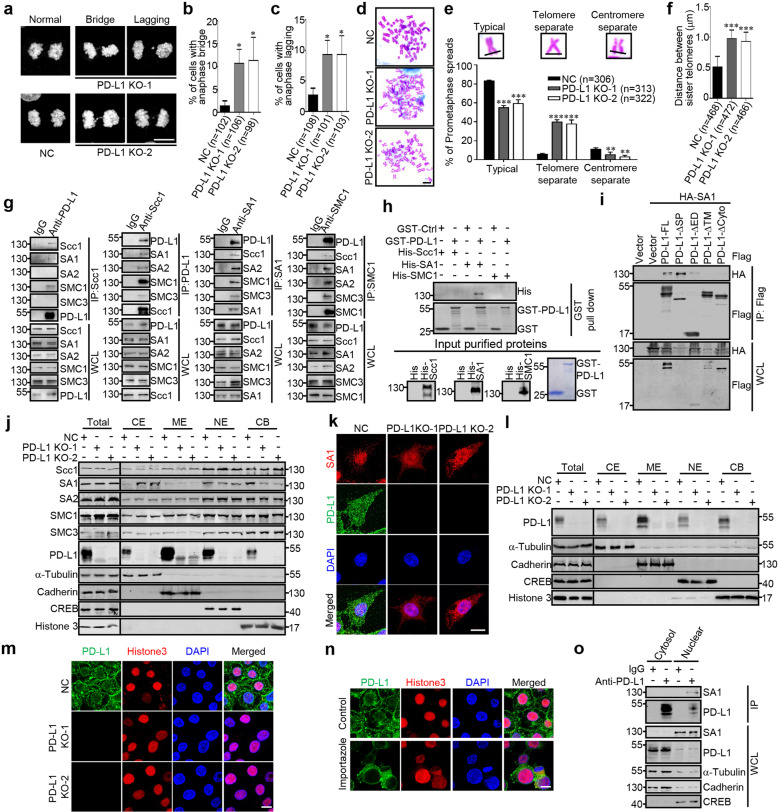
**a–c** Representative images of anaphase bridge and lagging chromosomes in negative control (NC) and PD-L1 knockout (KO) RKO cells **a**. The percentage of cells with anaphase bridge (**b**) and lagging (**c**) were counted. **d** The indicated cells were subjected to chromosome spread assay and followed by Giemsa staining after treatment with colcemid for 2.5 h. Scale bar: 10 μm. **e** Graphical representation of the frequency of each type of chromosome morphology. The classification was assigned when five or more chromosomes in a spread displayed the indicated morphology. The cells with different chromosomal morphology were counted. **f** Graphical representation of the distances between sister telomeres and the length was determined by Image J software. **g** Total lysates from RKO cells were subjected to co-IP analyses with antibodies against PD-L1, Scc1, SA1, or SMC1, respectively. **h** In vitro interactions of purified GST-PD-L1 with His-Scc1, His-SA1, and His-SMC1. Coomassie staining was used to visualize GST or His fusion proteins. Western analysis with anti-His was used to visualize His-fusion proteins. **i** HEK293T cells transfected with the indicated plasmids were used for co-IP experiments. **j** Fractionations of subcellular proteins form NC and PD-L1 KO RKO cells were analyzed by western blotting using the indicated antibodies. CE cytoplasmic extract, ME membrane extract, NE nucleoplasm extract, CB chromatin-bound nuclear extract. **k** Immunofluorescence analyses were carried out by using anti-PD-L1 and anti-SA1 antibodies. Scale bar, 10 μm. **l** Fractionations of subcellular proteins form NC and PD-L1 KO RKO cells were analyzed by western blotting using the indicated antibodies. **m** Immunostaining analyses were carried out by using anti-PD-L1 and anti-SA1 antibodies. Scale bar, 10 μm. **n** Wild-type RKO cells were treated with or without Importazole (1 μM) for 3 h, and subjected immunofluorescence assay with anti-PD-L1 and anti-Histone3 antibodies. Scale bar, 10 μm. **o** Wild-type RKO cells were isolated to cytosol (containing cytoplasmic and membrane proteins) and nuclear fractions, and processed for co-IP assay with anti-PD-L1 antibody. Quantitative data from at least three independent experiments are shown as the mean ± SD. The sample size (*n*) is indicated. ^∗^*P* < 0.05, ^∗∗^*P* < 0.01 and ^∗∗∗^*P* < 0.001, Student’s *t*-test

Next, we aimed to uncover the potential mechanism by which PD-L1 regulates genomic stability. We found that endogenous PD-L1 interacts with Scc1, SA1, and SMC1 in RKO cells (Fig. 1g). In vitro pull-down assay showed that GST-PD-L1 can directly interact with His-SA1, but not His-Scc1/His-SMC1 (Fig. 1h). We also delineated the region of PD-L1 responsible for its interaction with SA1 by constructing a series of deletion mutants. PD-L1 protein contains a signal peptide (SP), a large extracellular domain (ED), a transmembrane domain (TM), and a cytoplasmic tail (Cyto) (Supplementary Fig. S2). The co-IP data showed that deletion of TM or Cyto domain of PD-L1, but not its SP or ED domain, abolished its interaction with SA1 (Fig. 1i). As the signal peptide is not required for its interaction with SA1, suggesting that PD-L1 may interact with SA1 independent of its membrane-localization. Depletion of TM may secret PD-L1 out of the cells. Thus, we conclude that the Cyto domain of PD-L1 is essential for its interaction with SA1.

Depletion of either PD-L1 or SA1 siRNAs in HeLa cells caused aneuploidy, abolished telomere cohesion without changing centromere cohesion, and significantly decreased the telomere length and suppressed cell proliferation (Supplementary Fig. S3). Importantly, double knockdown of PD-L1 and SA1 did not show synergistic effects compared with that of SA1 depletion (Supplementary Fig. S3), suggesting that PD-L1 and SA1 may act in the same pathway to regulate genomic stability. What’s more, our data showed that wild-type, but not PD-L1-ΔCyto, can rescue the genomic instability phenotypes and cancer cell proliferation defects caused by PD-L1 depletion, suggesting that binding of PD-L1 to SA1 via its cytosolic tail is required for PD-L1-mediated genomic stability (Supplementary Fig. S4).

Next, deletion of PD-L1 had no significant effect on either total protein or mRNA levels of all cohesin subunits (Fig. 1j; Supplementary Fig. S5), but the proteins levels and cellular distribution of SA1 were apparently reduced in the nucleus, while increased in cytoplasm (Fig. 1j, k). As a control, we did not detect any apparent effect on the subcellular distributions of other cohesin components (Fig. 1j). Consistently, we found that WT, but not PD-L1-ΔCyto, can restore the nuclear localization of SA1 (Supplementary Fig. S6), suggesting their interaction is required for PD-L1-mediated SA1 subcellular redistribution. Just recently, Yu et al. also discovered the function of nuclear PD-L1 in regulation of cohesion through cohesin regulatory subunits—PDS5B, WAPL, and Sororin,^4^ while we found that PD-L1 directly binds to SA1, which may be another mechanism that exists in different cell types.

Although PD-L1 could be detected both in the plasma membrane and nucleus, the nuclear localization of PD-L1 is still debated. Therefore, we first verified whether PD-L1 is indeed able to be detected in the nucleus. We performed cellular fraction and immunofluorescence assays, and confirmed that PD-L1 was highly distributed in the cell membrane, and we also found a considerable amount of PD-L1 located in cytoplasm, nucleoplasm, and chromatin-bound nuclear extract (Fig. 1l, m). Our data clearly showed that PD-L1 could indeed be detected in nucleus (Fig. 1m).

To further reveal the mechanism of PD-L1 nuclear translocation, we inhibited the potential nuclear import by an importin blocker, importazole. Our results showed that importazole treatment significantly reduced the nuclear accumulation of PD-L1 (Fig. 1n). To further explore the mechanism of importin-mediated PD-L1 nuclear distribution, we screened a panel of importinα subfamily including karyopherin (KPNA)1–6 and found that only KPNA2 and KPNA6 markedly bind to PD-L1 (Supplementary Fig. S7a). Moreover, knockdown either of KPNA2 or KPNA6 reduced nuclear translocation of PD-L1 in RKO cells (Supplementary Fig. S7b–e). Taken together, these data suggest the essential roles of KPNA2 and KPNA6 in nuclear translocation for PD-L1. Lately, Gao et al. reported that KPNA2 interacts with PD-L1 and mediates its nuclear translocation, which confirmed our findings.^5^ Further data showed that nuclear, but not cytoplasm/membrane-localized PD-L1 significantly interacted with SA1 (Fig. 1o).

As *PD-L1* is highly expressed in various cancers, we analyzed whether expression of *SA1* is correlated with *PD-L1* in cancer tissues. Data from datasets of TCGA showed that expression of *PD-L1* is positively correlated with *SA1* in several types of cancer patients, such as colon cancer, breast invasive carcinoma, and so on (Supplementary information, Fig. S8), suggesting that PD-L1 is a potential regulator of SA1 in tumorigenesis.

Collectively, our results demonstrated that knockout of PD-L1 impairs the subcellular distribution of SA1 and provokes chromosome segregation errors resulting in telomere cohesion dysfunction and aneuploidy. Moreover, we provided evidence showing that PD-L1 directly binds to cohesin-SA1 via its cytosolic tail in the nucleus. In addition, PD-L1 is imported to the nucleus via an importin-dependent pathway. Thus, our work suggests that nuclear PD-L1 associates with cohesin-SA1 to reinforce mitotic surveillance against genomic instability in cancers.

## Supplementary information

Supplementary Materials
