# Supplementary material for: PD-L1 regulates genomic stability via interaction with cohesin-SA1 in the nucleus
Source: Signal Transduct Target Ther. 2021 Feb 25;6:81. doi: 10.1038/s41392-021-00463-0 (PMC7904913; doi:10.1038/s41392-021-00463-0)
Supplement: Supplementary file 1 — Supplementary Materials [file 41392_2021_463_MOESM1_ESM.docx]

Supplementary Materials for

PD-L1 regulates genomic stability via interaction with Cohesin-SA1 in the Nucleus

Wen Zhang^1,7^, Jiali Jin^1,7^, Yanjin Wang^1,7^, Lan Fang^1^, Liu Min^4^, Xinbo Wang^1^, Lin Ding^2^, Linjun Weng^2^, Tan Xiao^1^, Tianhua Zhou^4,5,6^, Ping Wang^1^

1. Tongji University Cancer Center, Shanghai Tenth People's Hospital of Tongji University, School of Medicine, Tongji University, Shanghai 200092, China

2. School of Life Sciences and Technology, Tongji University, Shanghai 200092, China

4. Department of Cell Biology and Cancer Institute of the Second Affiliated Hospital, Zhejiang University School of Medicine, Hangzhou 310058, China

5. Cancer Center, Zhejiang University, Hangzhou 310058, China

6. Department of Molecular Genetics, University of Toronto, Toronto, ON M5S 1A8, Canada

7. These authors contributed equally:

Correspondence to: wangp@tongji.edu.cn

**This PDF file includes:**

Materials and Methods

Supplementary Text

Figures. S1 to S8

Materials and Methods

**Plasmids**

pCMV-3’HA was used for PD-L1 and SA1 expression plasmids. PCDNA3.1-4Flag vector was used for KPNAs expression plasmids. GST-tagged PD-L1 was cloned into pGEX-4T-2. His-tagged cohesin proteins were cloned into pFastBacHT A. The lentiviral vector lentiCRISPRv2 carrying both Cas9 enzyme and a gRNA targeting PD-L1. Construction of PD-L1 deletion mutants was based on PD-L1 full-length plasmid which contains C-terminal Flag Tag. The PD-L1-ΔSP mutant deleted the amino acids (aa) 1-18, the PD-L1-ΔED mutant deleted the amino acids 19-238, the PD-L1-ΔTM mutant deleted the amino acids 239-259, the PD-L1-ΔCyto mutant lacking the amino acids 260-290 were generated by PCR (Supplementary information, Fig S2). PD-L1 RNAi-resistant mutants were constructed by site mutation for rescue expression in PD-L1 knockdown cell. All these constructs were confirmed by DNA sequencing.

gRNA sequences:

PD-L1 gRNA-1: 5’-CACCGACTGCTTGTCCAGATGACTT-3’

PD-L1 gRNA-2 5’-CACCGGTTCCCAAGGACCTATATG-3’

**Antibodies and Reagents**

The following antibodies were purchased from the indicated suppliers：PD-L1 (Cell Signaling Technology, 13684), SA1 (Proteintech, 14015-1-AP), SA2 (Proteintech, 19837-1-AP), SCC1 (Proteintech, 27071-1-AP), SMC1 (Proteintech, 21695-1-AP), SMC3 (Proteintech, 14185-1-AP), CREB (Cell Signaling Technology, 9197) pan-cadherin (Diagbio, db4569), Histone3 (Cell Signaling Technology, 4499), KPNA2 (Proteintech, 10819-1-AP), KPNA6 (Proteintech, 12366-2-AP), alpha-Tubulin (Proteintech, 11224-1-AP), Flag antibody (Diagbio, db7002), HA antibody (Diagbio, db2603).

Importazole was purchased from MedChem Express (Cat. no. HY-101091). Lipofectamine RNAiMAX Reagent was purchased from ThermoFisher (Cat. no. 13778). Lipofectamine 2000 was purchased from ThermoFisher (Cat. no. 18324010).

**Cell Culture and transfection**

RKO, HEK293T and HeLa cells were cultured in DMEM with 10% fetal bovine serum at 37 °C in 5% CO2. Plasmids was transfected by Lipofectamine 2000 (ThermoFisher). siRNA was transfected by Lipofectamine RNAiMAX (ThermoFisher) according to the manufacturer's instructions.

siRNA sequences:

PD-L1 siRNA: 5’-GCCGAAGUCAUCUGGACAATT-3’

SA1 siRNA: 5’-GUGAUGCCUUCCUAAAUGATT-3’

KPNA2 siRNA-1: 5’-GCUCCUGCAUCAUGAUGAU-3’

KPNA2 siRNA-2: 5’-AUCAUCAUGAUGCAGGAGC-3’

KPNA6 siRNA-1: 5’-GGGAAAGACAAUUAUCGAA-3’

KPNA6 siRNA-2: 5’-GCUCUAAACCCUGAAGAAA-3’

**Lentiviral Production**

The lentiviral vectors were transfected into HEK293T cells together with the packaging plasmids psPAX2 and pMD2.G at the ratio of 4:2:1. The supernatant medium containing lentiviruses was collected 48h later for further use.

**Generation of PD-L1 knockout cells**

RKO cells were infected by the lentiviruses medium with polybrene (8 μg/ml). 48 h after infection, the cells were selected with puromycin (2 μg/mL) for one week. PD-L1 knockout cells were sorted by flow cytometer (BD FACS AriaII) using anti-PD-L1 antibody (Biolegend, 329706). Sorted cells were further confirmed by western blot.

**Real-Time Quantitative PCR**

Total RNAs were extracted by Trizol (Takara) according to the manufacturer’s instruction. RNA was reverse-transcribed into cDNA with the reverse transcription PCR MIX (Takara). Quantitative PCR was performed with SYBR qPCR Master Mix (Vazyme) and quantified by the CFX96 Real-Time PCR System (Bio-Rad).

qPCR primers:

Scc1-F: 5’-GGATAAGAAGCTAACCAAAGCCC-3’

Scc1-R: 5’-CTCCCAGTAAGAGATGTCCTGAT-3’

SA1-F: 5’-TGGCAGCGAGCTTGAAGAAA-3’

SA1-R: 5’-CCACCTCAAATAATGTGACAGGC-3’

SA2-F: 5’-TCCTTCTGGTCCAAACCGAAT-3’

SA2-R: 5’-ACCGACTGCATAGCACTCTTG-3’

SMC1-F: 5’-GGCAAAGGTACGGTCCTCAG-3’

SMC1-R: 5’-GGCAAAGGTACGGTCCTCAG-3’

SMC3-F: 5’-ATTGGTGCCAAAAAGGATCAGT-3’

SMC3-R: 5’-GATTGCTTCGAGAAAAACCAGC-3’

TEL-F: 5’-ACACTAAGGTTTGGGTTTGGGTTTGGGTTTGGGTTAGTGT-3’

TEL-R: 5’-TGTTAGGTATCCCTATCCCTATCCCTATCCCTATCCCTAACA-3’

Alb-F: 5’-GGCGGCGGGCGGCGCGGGCTGGGCGGAAATGCTGCACAGAATCC-3’

Alb-R: 5’-GCCCGGCCCGCCGCGCCCGTCCCGCCGGAAAAGCATGGTCGCCT-3’

GAPDH-F: 5’-GAGCGAGATCCCTCCAAAAT-3’

GAPDH-R: 5’-GGCTGTTGTCATACTTCTCATGG-3’

**Co-immunoprecipitation and western blotting**

For co-precipitation of Flag or HA-tagged proteins, cells transfected with the Flag or HA vectors were lysed in lysis buffer (50 mM Tris-HCl, pH 7.4, 150 mM NaCl, 10% glycerol, 1 mM EDTA, 0.5% Nonidet P-40 and a mixture of protease inhibitors) and subjected to immunoprecipitation with anti-Flag beads (Sigma-Aldrich) or anti-HA beads (Abmart) at 4 °C for 4 h. The immunoprecipitates were washed and then processed for western blotting.

For endogenous protein coimmunoprecipitation, cell lysates were incubated with indicated antibodies at 4 °C overnight and added protein A/G Sepharose beads (Santa Cruz) for another 4 h. 5% whole cell lysate were loaded as input sample. The immunoprecipitates were washed and then processed for western blotting.

For cytosolic and nuclear protein coimmunoprecipitation, the cells were lysed with buffer A (10 mM HEPES [pH 7.9], 1.5mM MgCl2, 10 mM KCl, 0.5 mM DTT, 0.05% NP40 and protease inhibitor cocktail). After 15 min on ice, the cells were centrifuged at 3,000 rpm and 4°C for 10 min. The resultant supernatant was then used as a cytosolic fraction. The pellet was then homogenized with buffer B (20 mM HEPES [pH 7.9], 0.4 M NaCl, 1 mM EDTA, 1 mM DTT, and 1 mM PMSF). After 10 min of vigorous vortexing, the homogenates were centrifuged for 20 min at 13,000 rpm and 4°C. The resultant supernatants were then used as nuclear fractions. The nuclear and cytosolic fractions were quantified using the Bradford method and for endogenous protein immunoprecipitations.

For western blotting, the protein samples were separated by SDS-PAGE gel, transferred to nitrocellulose blotting membrane (GE), incubated with indicated antibodies and detected by ChemiDoc Touch Imaging System (Bio-Rad) or LI-COR Odyssey imaging systems (LI-COR).

**GST Pull-down**

His-tagged SCC1, SA1 and SMC1 were produced in insect Sf9 cells with infection of recombinant baculovirus for 72h according to Bac-to-Bac baculovirus expression system. GST was purified in E. coli BL21 (DE3) cells using glutathione-agarose beads. GST-PD-L1 generated in E. coli BL21 (DE3) cells was expressed as insoluble inclusion bodies with denaturation, refolding and dialysis. To detect the association among these proteins, the blots were probed with the respective antibodies as indicated.

**Immuno**[**fluorescence**](javascript:;)

Cells grown on glass coverslips were fixed with 4% paraformaldehyde for 15 min, and then incubated with primary antibodies overnight at 4°C and secondary antibodies for 1 h at room temperature. DNA was labelled by DAPI (Sigma). The sections were moved flatly to a microscope slide after staining. The mounted coverslips were analyzed by confocal microscopes (Zeiss LSM880 microscopy or Nikon Eclipse Ti-U confocal microscopy).

**Subcellular Fractionation**

For cytoplasmic, membrane, [nucleoplasm](javascript:;) and chromatin-bound nuclear extract, cells were fractionated using Subcellular Protein Fractionation Kit for Cultured Cells (78840, Thermo Fisher Scientific) according to manufacturer’s instruction.

**Chromosome spread assays**

Chromosome spread assays for Giemsa staining were prepared as previously described. In brief, cells were treated with 100 ng/ml of colcemid (477-30-5, Sigma) for 2.5 h, and mitotic cells were collected by shaking-off. Cells were incubated in a hypotonic solution (DMEM: H_2_O at a ratio of 2:3) for 5.5 min at room temperature and fixed with freshly prepared Carnoy’s solution (methanol: acetic acid at a ratio of 3:1). Cells in Carnoy’s solution were dropped onto glass slides, stained with 5% Giemsa (G1015, Solarbio) and analyzed by bright field microscopy (BX81, Olympus).

**Quantification and Statistical Analysis**

All experiments were repeated at least three times and data were shown as mean ± SD. Statistical significance was evaluated by Student *t*-test. *P* value was considered statistically significant. In the graphed data ∗, ∗∗ and ∗∗∗ denote *P* values of < 0.05, 0.01 and 0.001, respectively. ns, not significant.

Supplementary Text

Acknowledgments

We thank Dr. Yuehong Yang for kindly providing His-tagged SCC1, SA1 and SMC1 plasmids, Yaxu Li, Tong Meng, Yue Liu for their discussions and comments on the manuscript. We also thank the members of the Wang lab for their assistance. This work was supported by the National Natural Science Foundation of China (81625019, 31830053, 31920103007, 31701214, 31801178, 31871398, 31900525), the China Postdoctoral Science Foundation (2019M650090, 2019T120353), the Science Technology Commission of Shanghai Municipality (18410722000), Shanghai Youth Science and Technology Star Program (19QA1406700), the “Chenguang Program” supported by Shanghai Education Development Foundation and Shanghai Municipal Education Commission (19CG20), the Shanghai Sailing Program (18YF1419300).

Author contributions

W.Z., J.J., Y.W., T.Z., and P.W. conceived the project and designed experiments. W.Z., J.J., Y.W., L.F., M.L., L.D. and L.W., performed experiments. W.Z., J.J., X.W. and P.W. analyzed the data. W.Z., J.J., L.F., and P.W. wrote the manuscript.

Competing interests: The authors declare no competing of interests.

Figure. S1.


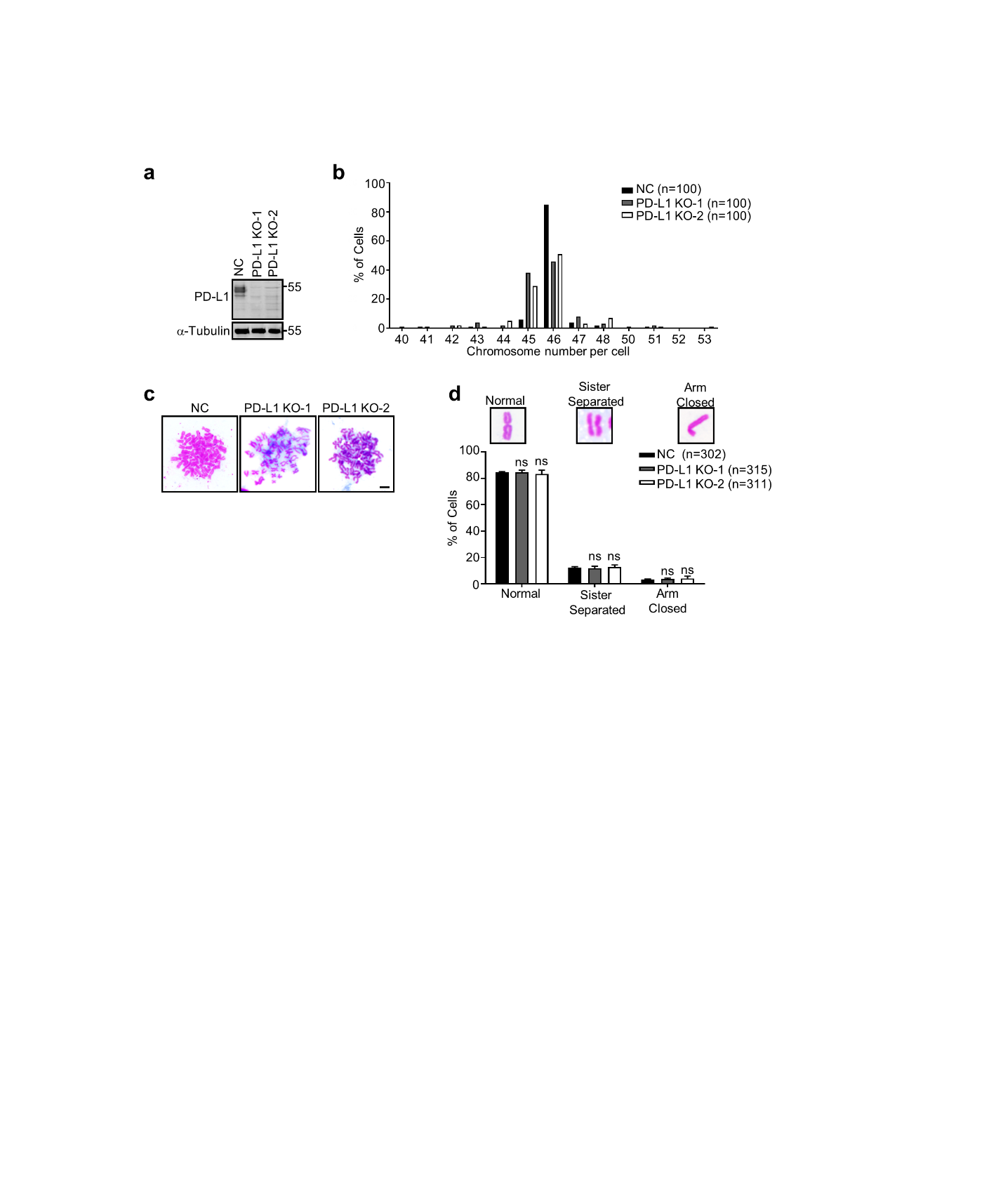


**PD-L1 depletion causes aneuploidy but not centromere cohesion defects.** Control (NC) and PD-L1 KO (PD-L1 KO-1 and PD-L1 KO-2) RKO cells were processed for the following analyses. **a** Immunoblot results showed knockout efficiency of PD-L1. **b** Graph showing the distribution in the number of chromosomes of 100 metaphases from each group. **c** The indicated cells were subjected to chromosome spread assay and followed by Giemsa staining after treatment with colcemid for 2.5 h. Scale bar: 10 μm. **d** Graphical representation of the frequency of each type of chromosome morphology. The classification was assigned when five or more chromosomes in a spread displayed the indicated morphology. The cells with different chromosomal morphology were counted. Quantitative data from at least three independent experiments are shown as the mean ± SD. More than 100 cells are calculated for each group. ns, not significant (*P* > 0.05), Student’s *t*-test.

Figure. S2.


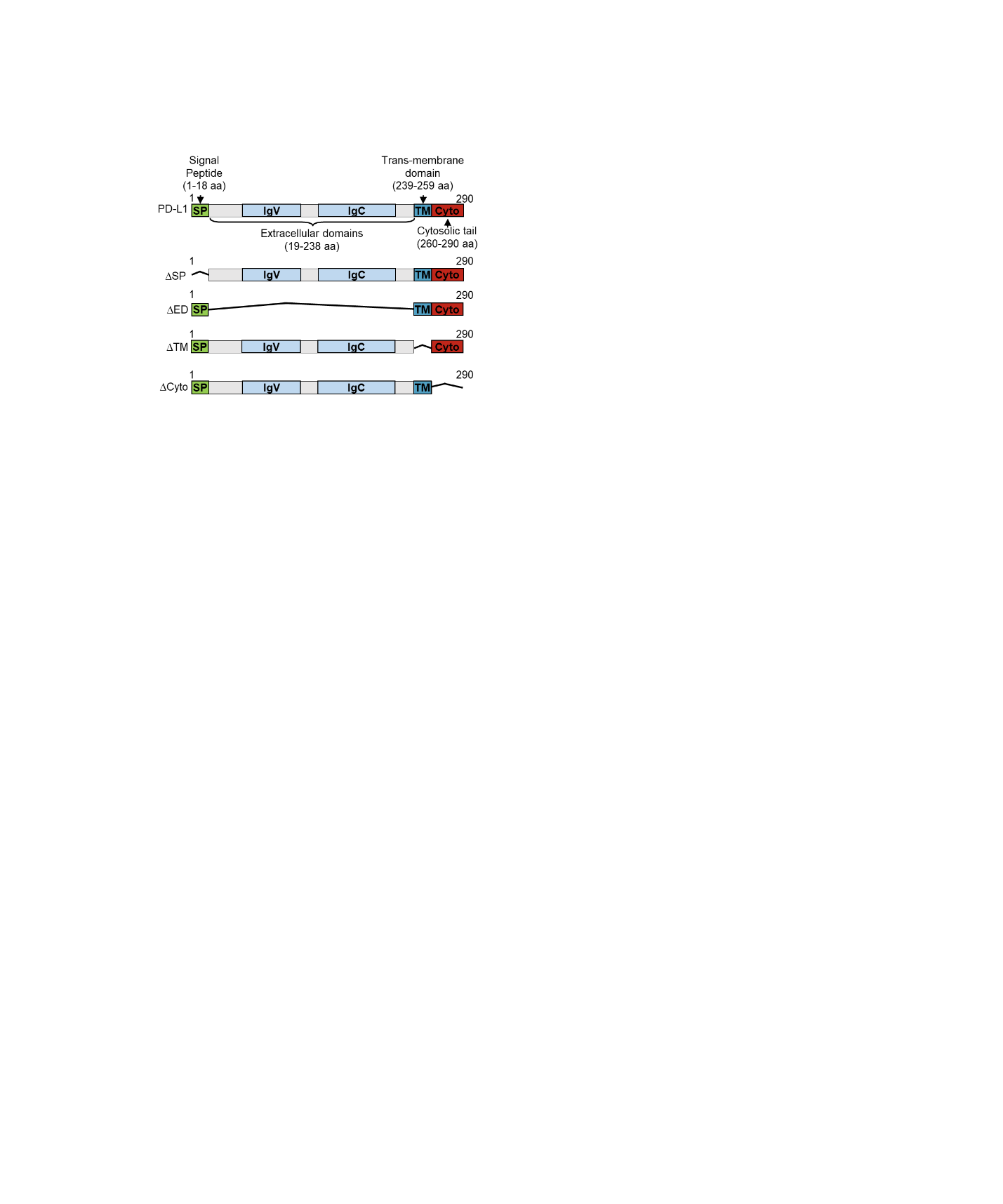


**Schematic diagram of PD-L1 domains.** Construction of PD-L1 deletion mutants was based on PD-L1 full-length plasmid. The PD-L1-ΔSP mutant deleted the amino acids (aa) 1-18, the PD-L1-ΔED mutant deleted the amino acids 19-238, the PD-L1-ΔTM mutant deleted the amino acids 239-259, and the PD-L1-ΔCyto mutant lacking the amino acids 260-290.

Figure. S3.


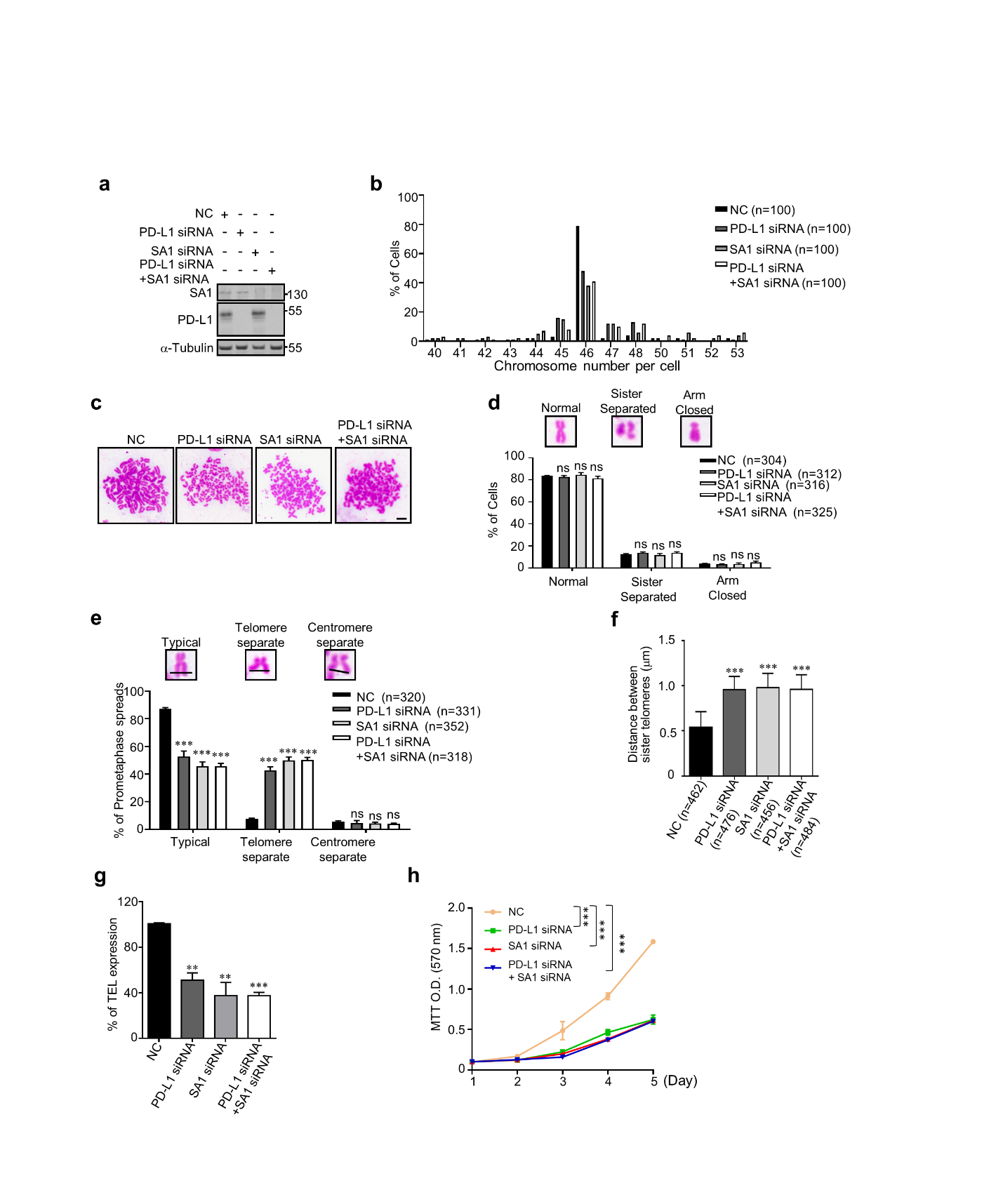


**PD-L1 and SA1 act in the same pathway to regulate genomic stability.** HeLa cells treated with the indicated siRNAs were processed for the following analyses. **a** Immunoblot results showed knockdown efficiency of PD-L1 and SA1. α-Tubulin was used as a loading control. **b** Graph showing the distribution in the number of chromosomes of 100 metaphases from each group. **c** The indicated cells were subjected to chromosome spread assay and followed by Giemsa staining after treatment with colcemid for 2.5 h. Scale bar: 10 μm. **d, e** Graphical representation of the frequency of each type of chromosome morphology. The classification was assigned when five or more chromosomes in a spread displayed the indicated morphology. The cells with different chromosomal morphology were counted. **f** Graphical representation of the distances between sister telomeres and the length was determined by Image J software. **g** Relative telomere length of indicated cells. *Alb* was used as an internal control. **h** MTT assay was performed to measure the proliferation rates of indicated cells at different time points. Quantitative data from at least three independent experiments are shown as the mean ± SD. The sample size (n) is indicated. ***P* < 0.01 and ****P* <0.001, ns, not significant (*P* > 0.05), Student’s *t*-test.

Figure. S4.


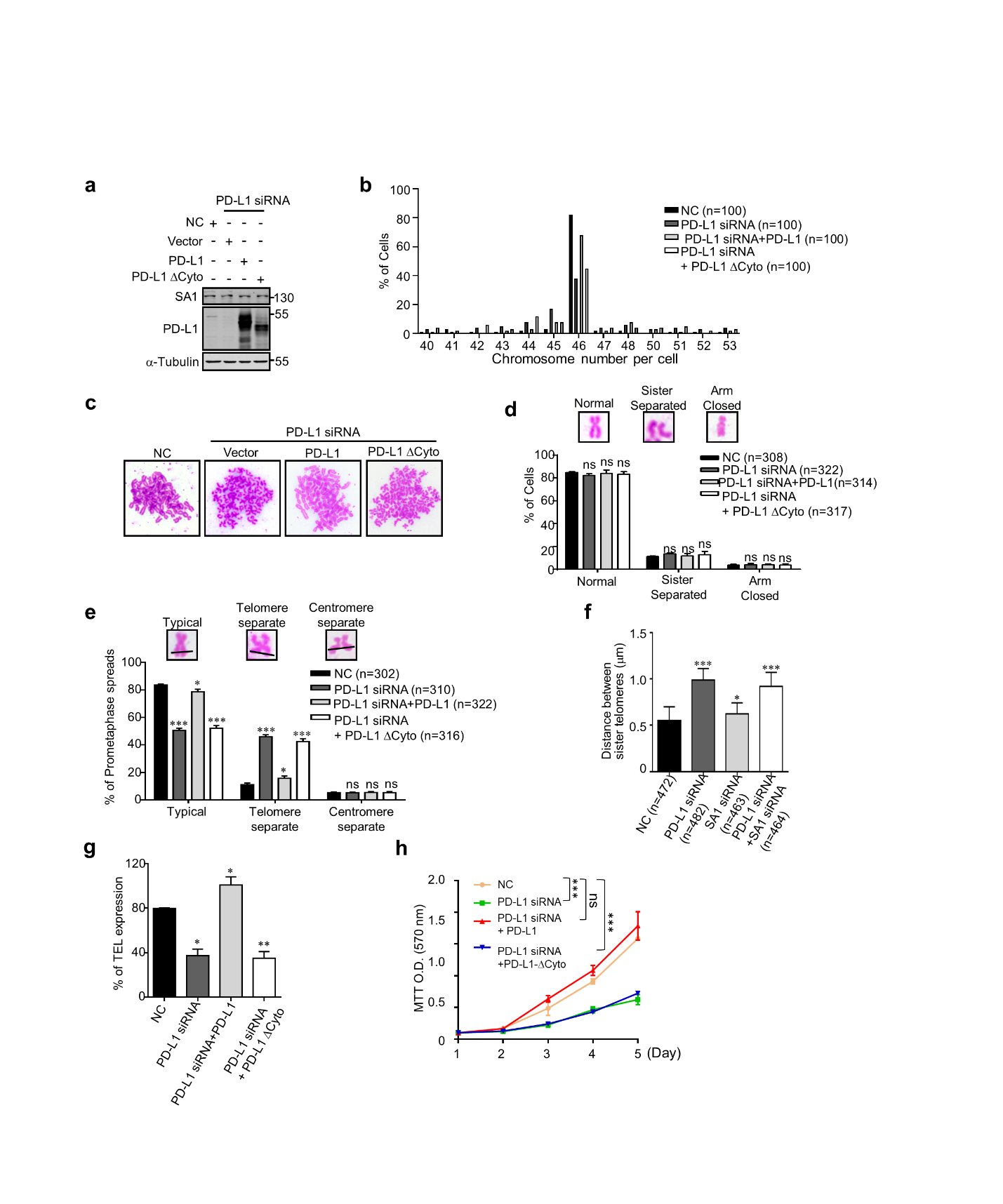


**PD-L1, but not PD-L1-ΔCyto, partially restores the nuclear localization of SA1 and rescues genomic instability phenotypes.** HeLa cells treated with the indicated siRNAs and plasmids were processed for the following analyses. **a** Immunoblot results showed expression of PD-L1 and SA1. α-Tubulin was used as a loading control. **b** Graph showing the distribution in the number of chromosomes of 100 metaphases from each group. **c** The indicated cells were subjected to chromosome spread assay and followed by Giemsa staining after treatment with colcemid for 2.5 h. Scale bar: 10 μm. **d, e** Graphical representation of the frequency of each type of chromosome morphology. The classification was assigned when five or more chromosomes in a spread displayed the indicated morphology. The cells with different chromosomal morphology were counted. **f** Graphical representation of the distances between sister telomeres and the length was determined by Image J software. **g** Relative telomere length of indicated cells. *Alb* was used as an internal control. **h** MTT assay was performed to measure the proliferation rates of indicated cells at different time points. Quantitative data from at least three independent experiments are shown as the mean ± SD. The sample size (n) is indicated. **P* < 0.05, ***P* < 0.01 and ****P* <0.001, ns, not significant (*P* > 0.05), Student’s *t*-test.

Figure. S5.


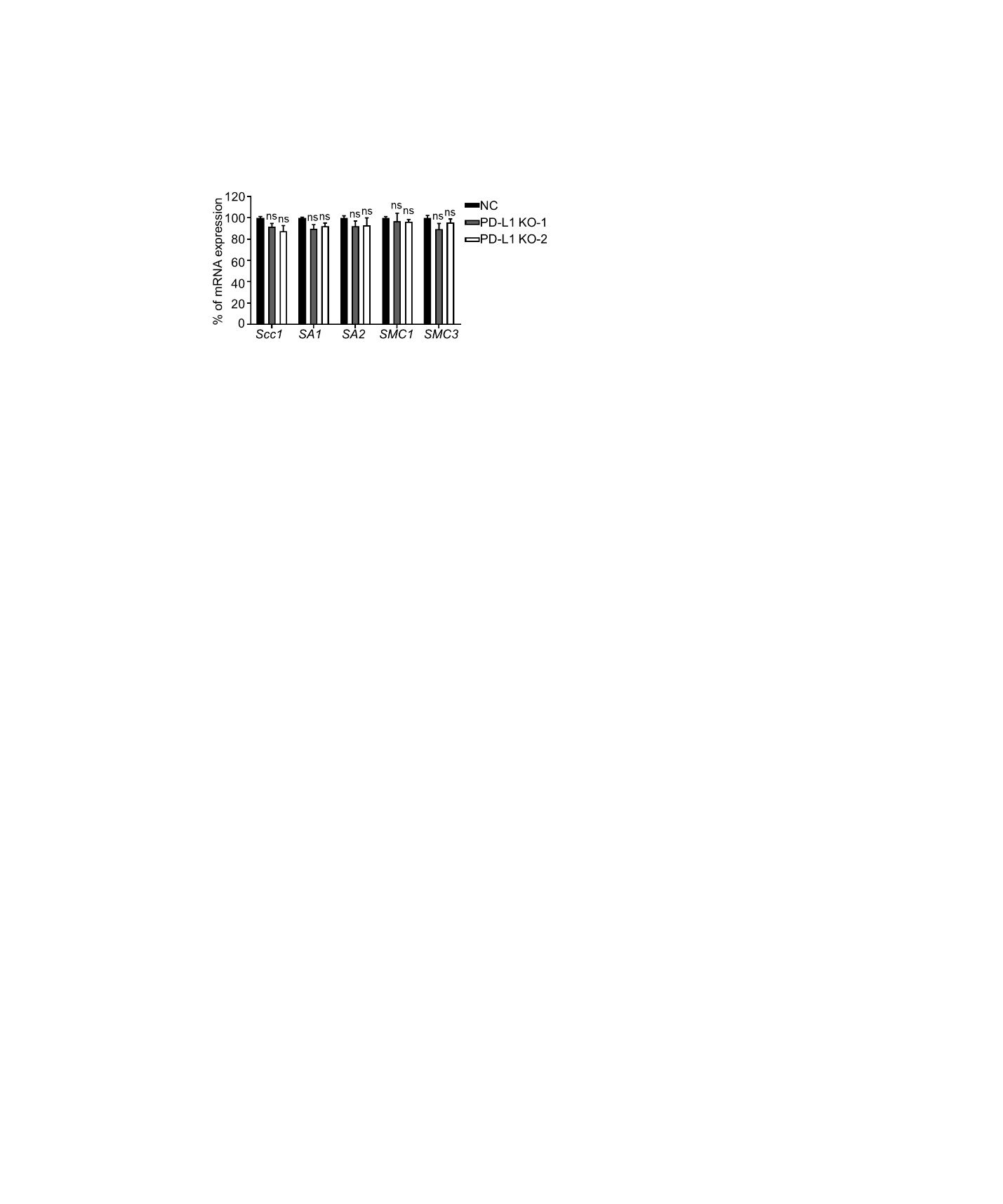


**PD-L1 has no significant effect on cohesin complex subunits mRNA expression.** The mRNA expression of cohesin complex subunits Scc1, SA1, SA2, SMC1, SMC3 in RKO wild-type and PD-L1 knockout cells. Quantitative data from at least three independent experiments are shown as the mean ± SD. ns, not significant (*P* > 0.05), Student’s *t*-test.

Figure. S6.


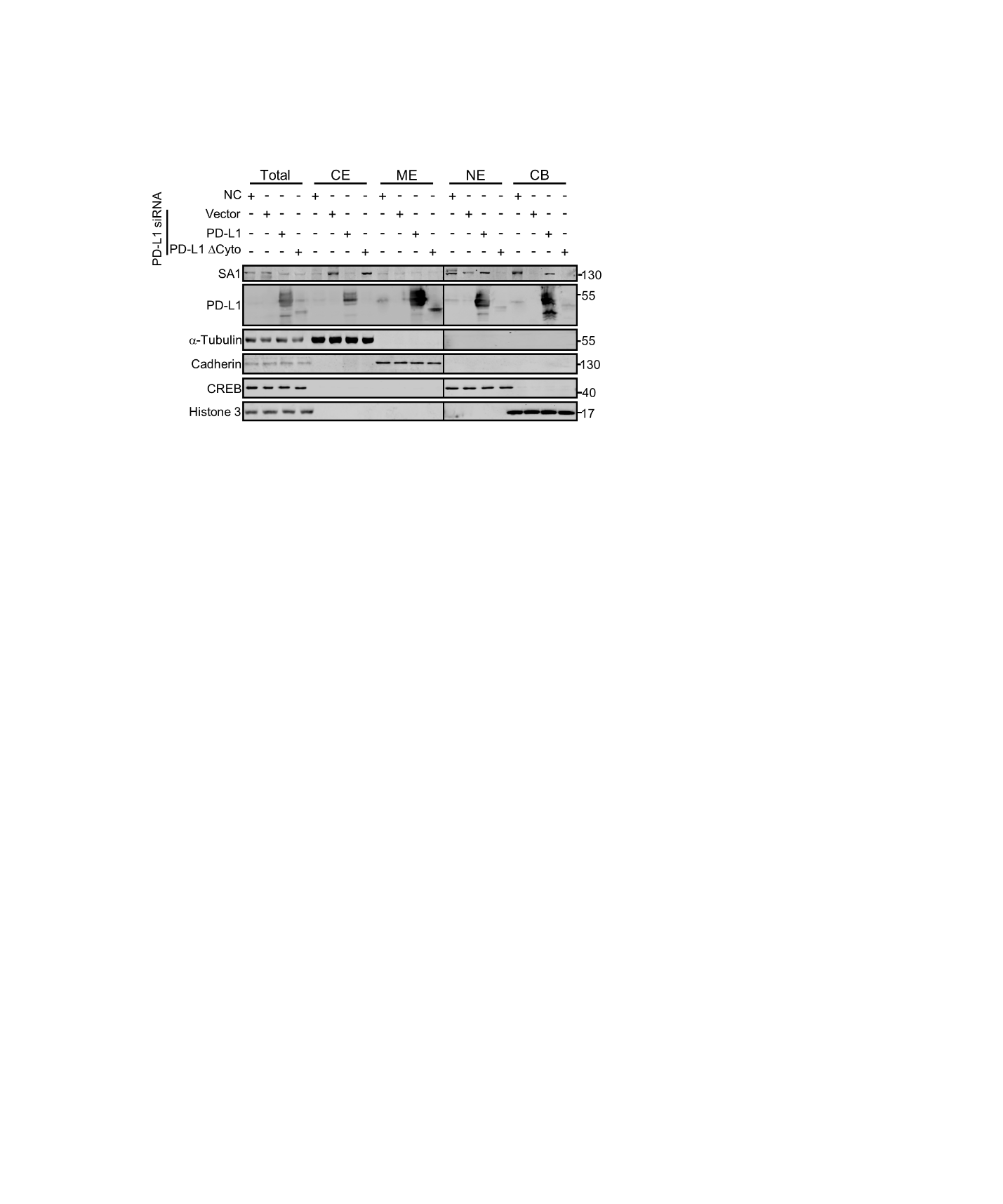


**Wild-type PD-L1 rescues the distribution defects of SA1 induced by PD-L1 depletion, but not its mutant.** Cell fraction assay showed full-length, but not PD-L1-DCyto decreased the protein level of cytoplasmic SA1, while increased SA1 protein level in the nuclear in HeLa cells.

Figure. S7.


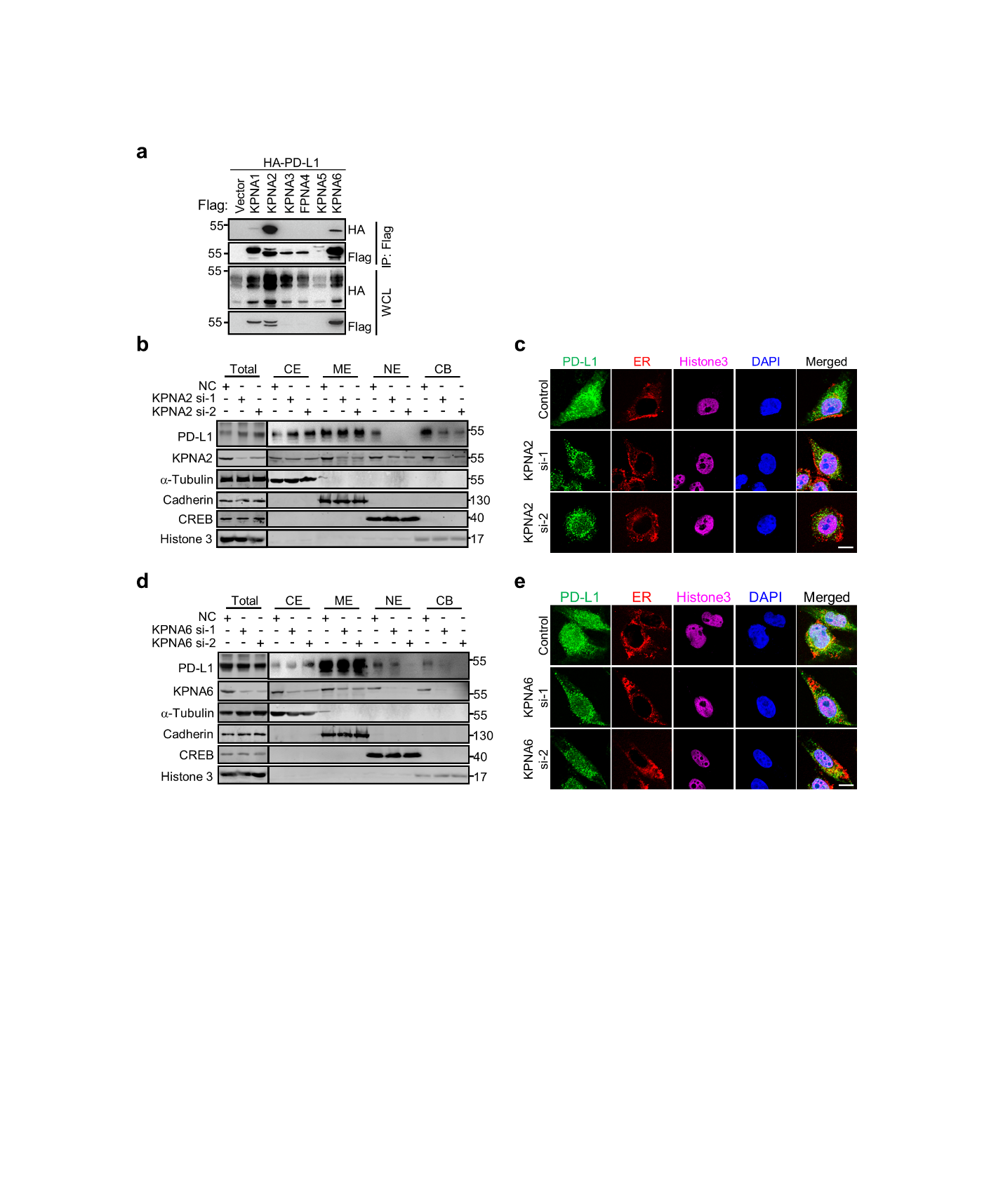


**The classical importins (KPNA2 and KPNA6) are required for the nuclear transport of PD-L1. a** co-IP screen the interaction of PD-L1 and importin a subfamily karyopherin (KPNAs) showed PD-L1 binds to KPNA2 and KPNA6. **b** KPNA2 knockdown partially blocked nuclear translocation of PD-L1 in RKO cells. **c** Immunofluorescence analysis of PD-L1 in RKO wild-type and KPNA2 knockdown cells. Scale bar, 10 μm. **d** KPNA6 knockdown partially blocked nuclear translocation of PD-L1 in RKO cells. **e** Immunofluorescence analysis of PD-L1 in RKO WT and KPNA6 knockdown cells. Scale bar, 10 μm.

Figure. S8.


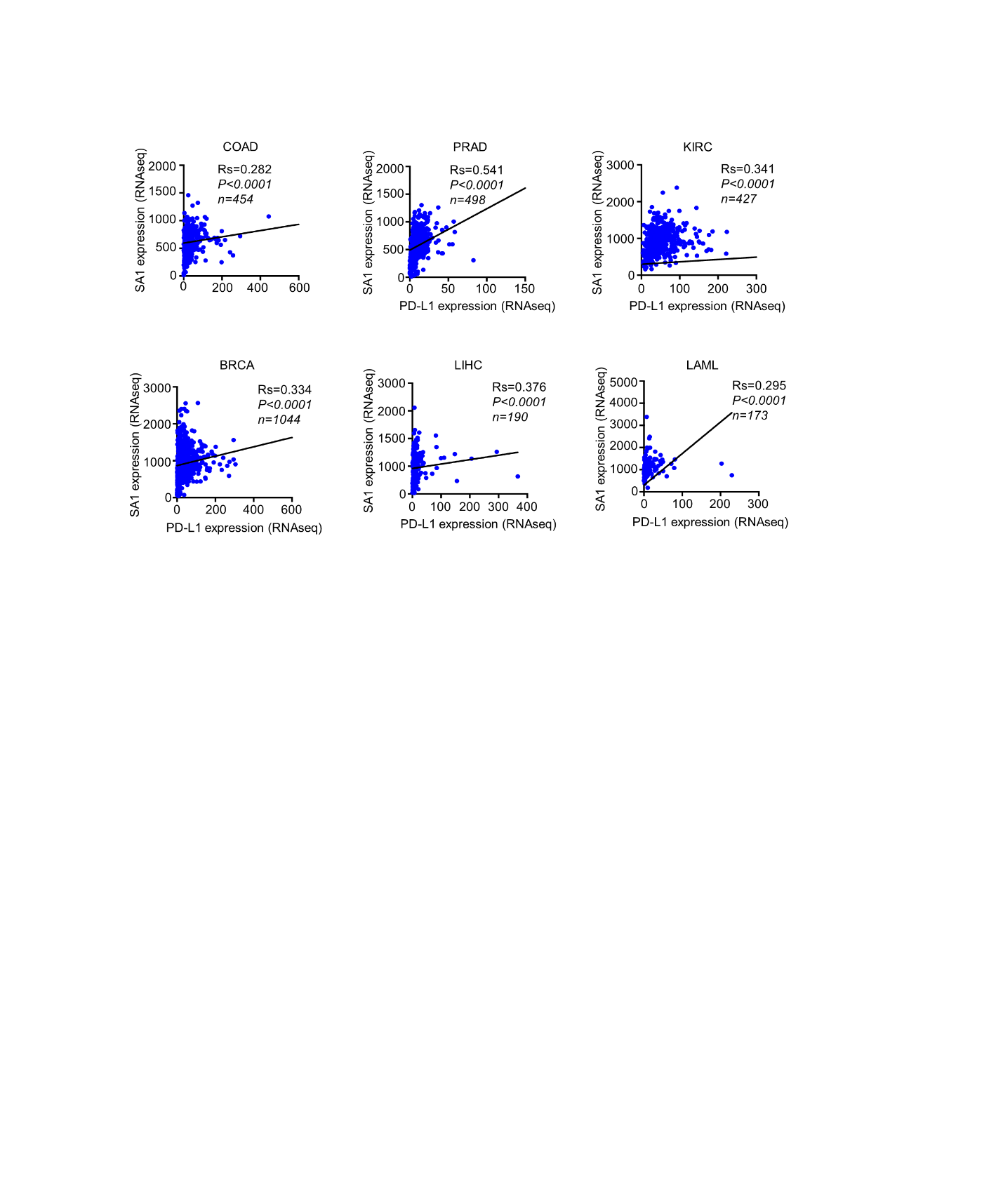


***PD-L1* gene expression is positively correlated with *SA1* in Cancers.** Colon adenocarcinoma (COAD), Prostate Cancer (PRAD), Kidney renal clear cell carcinoma (KIRC), Breast invasive carcinoma (BRCA), Liver hepatocellular carcinoma (LIHC) and Acute Myeloid Leukemia (LAML). Data is obtained from The Cancer Genome Atlas (TCGA). Gene expression correlation was assessed by the Spearman test between the indicated groups.
